# Supplementary material for: Mapping the Concept, Content, and Outcome of Family-Based Outdoor Therapy for Children and Adolescents with Mental Health Problems: A Scoping Review
Source: Int J Environ Res Public Health. 2022 May 10;19(10):5825. doi: 10.3390/ijerph19105825 (PMC9141554; doi:10.3390/ijerph19105825)
Supplement: Supplementary file 1 [file ijerph-19-05825-s001.zip › ijerph-1684227-supplementary.pdf]

International Journal of
Environmental Research and Public Health

MDPI

SUPPLEMENTARY FILES

1. Search strategy

AMED, EMBASE, MEDLINE & APA PsycInfo (Ovid): 19.11.2021. Database: AMED (Allied and Complementary Medicine) <1985 to November 2021>, Embase <1980 to 2021 Week 45>, Ovid MEDLINE(R) ALL <1946 to November 18, 2021>, APA PsycInfo <1806 to November Week 3 2021> Search Strategy. LINK

	#	Query	Results from 19 Nov 2021
**Search concept 1**		**Wilderness, outdoor therapies**	
Subject headings /	1	wilderness/ or Wilderness Experience/ or Adventure Therapy/ or Therapeutic Camps/ or wilderness therapy/	1,648
adj# = proximity / adjacency<br>ti = title words<br>ab = abstract words<br>kf = author words (MEDLINE, EMBASE)<br>id = concept (PscyInfo), subject supplements	2	((adventure* or outdoor* or nature* or wilderness* or recreation* or "open air" or natural or forest* or hiking or camp*) adj3 (therap* or treatment* or program* or rehab* or training or counselling or counseling or pscyhotherap*)).ti,ab,kf,id.	58,459
	3	((adventure* or outdoor* or wilderness) adj3 intervention*).ti,ab,kf,id.	406
	4	exp forests/	48,260
	5	recreation therapy/	1,542
	6	"outdoor behavio* healthcare".ti,ab,kf,id.	43
	7	(forest bath* or shinrin-yoku).ti,ab,kf.	191
	8	(ecotherap* or "eco therap*" or "eco psychoterap*").ti,ab,kf,id.	99
	9	((green or blue) adj (therap* or psychotherap*)).ti,ab,kf,id.	168
**Wilderness therapies**	**10**	**or/1-9**	**108,629**
**Search concept 2**		**Parents or caregivers (family based)**	
.hw =subject heading words	11	(parent* or mother* or father* or family or families or caregiver* or "care giver*").ti,ab,hw,id,kf.	4,755,961
**Search concept 3**		**Children or younger people**	
	12	(child* or young* or youth* or kid or kids or girl or girls or boy or boys or adolescen*).ti,ab,kf,id,hw.	10,109,440
**Search concepts combined with AND**	**13**	**10 and 11 and 12**	**3,515**
	14	*remove duplicates from 13*	*2,536*
Segments for EndNote exports	15	limit 14 to yr="2010 -Current"	1,487
	16	limit 14 to yr="2001-2009"	481
	17	limit 14 to yr="1860-2000"	568
	18	15 or 16 or 17	2,536

Notes

- Field codes: ti (words from title), ab (words from abstract), hw (words from subject headings), kf (author keywords, used in MEDLINE and EMBASE), id (concept (used in PsycInfo)
- Subject headings /
- Proximity/adjacency: adj#[1]
- Or/1-6 = 1 or 2 or 3 or 4 or 5 or 6

Total hits, date: 19.11.2021

13	10 and 11 and 12	**3515**
	AMED (Allied and Complementary Medicine) <1985 to November 2021>	31
	Embase <1980 to 2021 Week 45>	1483
	Ovid MEDLINE(R) ALL <1946 to November 18, 2021>	921
	APA PsycInfo <1806 to November Week 3 2021>	1080

[1] The ADJ operators finds two terms next to each other in the specified order.
The ADJ1 operators finds two terms next to each other in any order.
The ADJ2 operator finds terms in any order and with one word (or none) between them.
The ADJ3 operator finds terms in any order with two words (or fewer) between them.
The ADJ4 operator finds terms in any order and with three words (or fewer) between them, and so on
adj3 = N2 CINAHL (EBSCOhost), w/2 (Scopus)

CINAHL (EBSCO*host*) 22.11.2021

- Search modes - Boolean/Phrase/Advanced
- W# and N# = Proximities[2]
- MH = exact CINAHL subject headings
- The default fields for unqualified searches consist of the following: Title, Abstract and Subject headings[3]

#	**Query**	**Results**
S1	MH "Wilderness Experience"	280
S2	MH "Camping"	676
S3	(adventure* or outdoor* or nature* or wilderness* or recreation* or "open air" or natural or forest* or hiking or camp*) N2 (therap* or treatment* or program* or rehab* or training or counselling or counseling or pscyhotherap*)	8,327
S4	(adventure* or outdoor* or wilderness) N2 intervention*	74
S5	"outdoor behavio* healthcare"	12
S6	"forest bath*" or "shinrin-yoku"	37
S7	ecotherap* or "eco therap*" or "eco psychoterap*"	22
S8	(green or blue) W0 (therap* or psychotherap*)	283
S9	S1 OR S2 OR S3 OR S4 OR S5 OR S6 OR S7 OR S8	9,491
S10	parent* or mother* or father* or family or families or caregiver* or "care giver*"	609,934
S11	child* or young* or youth* or kid or kids or girl or girls or boy or boys or adolescen*	1,438,152
S12	*S9 AND S10 AND S11*	*676*

Scopus. 22.11.2021

Advanced search

TITLE-ABS-KEY(((adventure* OR outdoor* OR nature* OR wilderness* OR recreation* OR "open air" OR natural OR forest* OR hiking OR camp*) W/2 (therap* OR treatment* OR program* OR rehab* OR training OR counselling OR counseling OR pscyhotherap*)) OR ((adventure* or outdoor* or wilderness) w/2 intervention*) OR "outdoor behavio* healthcare" OR "forest bath*" or "shinrin-yoku" OR ecotherap* or "eco therap*" or "eco psychoterap*" OR ((green or blue) w/0 (therap* or psychotherap*))) AND TITLE-ABS-KEY (parent* or mother* or father* or family or families or caregiver* or "care giver*") AND TITLE-ABS-KEY(child* or young* or youth* or kid or kids or girl or girls or boy or boys or adolescen*)

Result: 1539 hits

[2] N# - Near Operator (N) - ex N5 finds the words if they are within five words of one another regardless of the order in which they appear. For example, type tax N5 reform to find results that would match tax reform as well as reform of income tax.
W# - Within Operator (W) - In the following example, W8 finds the words if they are within eight words of one another and in the order in which you entered them.

[3] CINAHL (EBSCOhost) guide http://support.ebsco.com/help/?int=ehost&lang=en&feature_id=Databases&TOC_ID=Always&SI=0&BU=0&GU=1&PS=0&ver=live&dbs=cin20jnh,cin20

Cochrane Central Register of Controlled Trials CENTRAL (Cochrane library)
Notes from Search help

- Proximity
    - NEAR: Finds the terms when they are within 6 words of each other. Terms can appear in either order. Example: cancer near lung (finds lung cancer as well as cancer of the lung)
    - NEAR/X: Finds the terms when they are within X words of each other where X = the maximum number of words between search terms. Terms can appear in either order. Example: cancer near/3 lung (finds lung cancer, as well as, cancer of the lung)
    - NEXT: Finds the terms when they appear next to each other. Terms must appear in the order specified. Use for phrase searching with wildcards. Example: lung next cancer (finds lung cancer but not cancer of the lung) . Example: hearing NEXT aid* (finds hearing aid and hearing aid
- Field codes:
    - Title Abstract Keyword: Default selection. Use this label to limit search to terms in the record title, abstract and keyword fields.
    - Keywords: Use this label to limit to keywords. Limit includes MeSH terms but does not allow for MeSH term explosion. Also searches EMBASE keyword fields

Advanced search, title, abstract and keywords:
wilderness OR "adventure therapy" OR camping OR "Recreation Therapy" OR ((adventure* or outdoor* or nature* or wilderness* or recreation* or "open air" or natural or forest* or hiking or camp*) NEAR/2 (therap* or treatment* or program* or rehab* or training or counselling or counseling or pscyhotherap*)) OR ((adventure* or outdoor* or wilderness) NEAR/2 intervention*) OR forest bath* or shinrin-yoku OR ecotherap* or "eco therap*" or "eco psychoterap*" OR ((green or blue) NEXT (therap* or psychotherap*)) OR forests OR (outdoor NEXT behavio* NEXT healthcare)
AND
parent* or mother* or father* or family or families or caregiver* or "care giver*"
AND
child* or young* or youth* or kid or kids or girl or girls or boy or boys or adolescen*

308 Trials matching
in Title Abstract Keyword - (Word variations have been searched)
Cochrane Central Register of Controlled Trials: Issue 10 of 12, October 2021

2. Reasons for exclusion after full text reading (n=51)

Language (n=1)	[1]
Wrong publication type (n=13)	[2]<br>[3]<br>[4]<br>[5]<br>[6]<br>[7]<br>[8]<br>[9]<br>[10]<br>[11]<br>[12]<br>[13]<br>[14]
No outdoor therapy (n=24)	[15]<br>[16]<br>[17]<br>[18]<br>[19]<br>[20]<br>[21]<br>[22]<br>[23]<br>[24]<br>[25]<br>[26]<br>[27]<br>[28]<br>[29]<br>[30]<br>[31]<br>[32]<br>[33]<br>[34]<br>[35]<br>[36]<br>[37]<br>[38]
Wrong population, lack of family involvement or not mental health outcome (n=13)	[39]<br>[40]<br>[41]

	[42]<br>[43]<br>[44]<br>[45]<br>[46]<br>[47]<br>[48]<br>[49]<br>[50]<br>[51]

Deviations from the protocol

Originally it was planned that two reviewers would independently pilot the data charting on a random sample of five included studies. Since the total inclusion of studies in this scoping review counted only two more (seven studies), it was for pragmatic reasons that one reviewer extracted all predefined data characteristics and that two additional reviewers verified these extracted data. Due to the limited number of included studies and the fact that these studies differed greatly regarding target populations and outcomes, it was deemed infeasible to develop a pattern chart according to the PAGER methodological framework [52]. Rather, it was decided to address the identified patterns, advances, and gaps in the literature in the discussion section.

REFERENCES SUPPLEMENTARY FILES:

1. Kondracka, J. Assumptions of therapeutic recreation and its role according to the research carried out in the vacation recreation centre Barretstown for children suffering from chronic and life threatening illnesses. *Psychiatria i Psychologia Kliniczna* **2013**, *13*, 58-66.
2. Alieva, T.; Mykhaylov, B.; Vashkite, I.; Vodka, M. Psychosocial rehabilitation of adolescents with deviant behavior. *European Psychiatry* **2015**, *30*, 1185.
3. Bettmann, J. Changes in adolescent attachment relationships as a response to wilderness treatment. *Journal of the American Psychoanalytic Association* **2007**, *55*, 259-265.
4. Birnbaum, A. Haven Hugs & Bugs. An innovative multiple-family weekend intervention for bereaved children, adolescents and adults. *The American journal of hospice & palliative care* **1991**, *8*, 23-29.
5. Canu, W.; Gordon, M. Mother nature as treatment for ADHD: overstating the benefits of green. *American journal of public health* **2005**, *95*, 371-372.
6. Cohen, C.; Zeitz, L. Our stories of collaboration throughout the therapeutic wilderness process. In *Family therapy with adolescents in residential treatment: Intervention and research.*, Christenson, J.D., Merritt, A.N., Eds.; Springer: 2017; pp. 93-108.
7. Cuines, K.; Center, B. Differential effects of an outdoor community-based youth development program on adolescent boys and girls. *Journal of Adolescent Health* **2010**, *46*, S73.
8. Freeman, P.A.; Zabriskie, R.B. The role of outdoor recreation in family enrichment. *Journal of Adventure Education and Outdoor Learning* **2002**, *2*, 131-145, doi:10.1080/14729670285200241.
9. Harper, N.; Cooley, R. Parental Reports of Adolescent and Family Weil-Being following a Wilderness Therapy Intervention: An Exploratory Look at Systemic Change. *Journal of Experiential Education* **2007**, *29*, 393-396.
10. Harper, N.J. Family Crisis and the Enrollment of Children in Wilderness Treatment. *Journal of Experiential Education* **2009**, *31*, 447-450.
11. Lakusta, C.; Wilkes, C.; Malhotra, S.; Vaz, G.; Cawthorpe, D. Adolescent day treatment program: Outcomes and follow-up study. *Eur. Psychiatry* **2012**, *27*, NOTE: CONFERANCE ABSTRACT only, doi:10.1016/S0924-9338(12)74473-5.
12. Lewicki, J.-A.; A, G.; K, M. Family camp: a multimodal treatment strategy for linking process and content. *J child youth care.* **1996**, *10*, 51-66.

13. Neff, J.M. Specialized summer camps provide benefits for children and families alike. *Exceptional Parent* **2009**, *39*, 34-36. Journal Article - pictorial.
14. Swartz, A. Camping as coping: children affected with HIV/AIDS spend a week in the woods. *HIV Impact* **2002**, 8-9.
15. Bachman, B. The development of a sustainable, community-supported children's bereavement camp. *Omega (United States)* **2013**, *67*, 21-35.
16. Bansal, P.S.; Waschbusch, D.A.; Haas, S.M.; Babinski, D.E.; King, S.; Andrade, B.F.; Willoughby, M.T. Effects of Intensive Behavioral Treatment for Children With Varying Levels of Conduct Problems and Callous-Unemotional Traits. *Behavior Therapy* **2019**, *50*, 1-14.
17. Bultas, M.W.; Budhathoki, C.; Balakas, K. Evaluation of child and parent outcomes after a pediatric cardiac camp experience. *Journal for Specialists in Pediatric Nursing* **2013**, *18*, 320-328.
18. Christian, D.D.; Perryman, K.L.; Portrie-Bethke, T.L. Improving the parent-adolescent relationship with adventure-based counseling: An Adlerian perspective. *Journal of Child and Adolescent Counseling* **2017**, *3*, 44-58.
19. Clark, J.; Kempler, H.L. Therapeutic family camping: A rationale. *The Family Coordinator* **1973**, *22*, 437-442.
20. Durkin, R. A competency-oriented summer camp and year-round program for troubled teenagers and their families. *Residential Treatment for Children & Youth* **1988**, *6*, 63-85.
21. Gibbs, A.; Moor, S.; Frampton, C.; Watkins, W. Impact of psychosocial interventions on children with disruptive and emotional disorders treated in a health camp. *Australian and New Zealand Journal of Psychiatry* **2008**, *42*, 789-799.
22. Greenfield, B.J.; Senecal, J. Recreational multifamily therapy for troubled children. *American Journal of Orthopsychiatry* **1995**, *65*, 434-439.
23. Griffiths, N.; Mazzucchelli, T.G.; Skinner, S.; Kane, R.T.; Breen, L.J. A pilot study of a new bereavement program for children: lionheart Camp for Kids. *Death studies* **2019**, 1-11.
24. Izenstark, D.; Oswald, R.F.; Holman, E.G.; Mendez, S.N.; Greder, K.A. Rural, Low-Income Mothers' Use of Family-Based Nature Activities to Promote Family Health. *Journal of Leisure Research* **2016**, *48*, 134-155, doi:10.18666/jlr-2016-v48-i2-6409.
25. Kiser, L.J.; Donohue, A.; Hodgkinson, S.; Medoff, D.; Black, M.M. Strengthening family coping resources: the feasibility of a multifamily group intervention for families exposed to trauma. *J. Trauma. Stress* **2010**, *23*, 802-806, doi:10.1002/jts.20587.
26. Kmita, G.; Baranska, M.; Niemiec, T. Psychosocial intervention in the process of empowering families with children living with HIV/AIDS - A descriptive study. *AIDS Care - Psychological and Socio-Medical Aspects of AIDS/HIV* **2002**, *14*, 279-284.
27. Kriel, C.; Schreck, C.M.; Watson, F. Participation of divorced single parents and their children in outdoor activities to improve attitudes and relationships. *S Afr J Res Sport Ph* **2016**, *38*, 69-79.
28. Lee, K.; Ewert, A. Adventure Programs and Diverse Family Styles. *Journal of Experiential Education* **2013**, *36*, 123-138, doi:10.1177/1053825913487886.
29. Lo, S.; Gidlow, B.; Cushman, G. Adventure Education and the Acculturation of First-Generation Chinese Canadians in Vancouver, Canada. *Journal of Experiential Education* **2014**, *37*, 113-128, doi:10.1177/1053825913489106.
30. Overholt, J.R. Role shifts and equalizing experiences through father-child outdoor adventure programs. *Leisure Sci* **2019**, No-Specified, doi:10.1080/01490400.2019.1627966.
31. Purvis, K.B.; Cross, D.R.; Federici, R.; Johnson, D.; McKenzie, L.B. The Hope Connection: A Therapeutic Summer Day Camp for Adopted and At-Risk Children with Special Socio-Emotional Needs. *Adoption and Fostering* **2007**, *31*, 38-48, doi:10.1177/030857590703100406.
32. Thomas, R. The Kinship Care Regional Project. *Developing Practice* **2010**, 55-64.
33. Tonkin, R. Camp Elsewhere: A summer program for adolescents with eating disorders. *Clin. Child Psychol. Psychiatry* **1996**, *1*, 597-606, doi:10.1177/1359104596014011.
34. Townsend, J.; Van Puymbroeck, M. Development and evaluation of a family recreation intervention for families with an adolescent with an autism spectrum disorder. *American Journal of Recreation Therapy* **2012**, *11*, 27-37, doi:10.5055/ajrt.2012.0032.
35. Townsend, J.A.; Van Puymbroeck, M. Parental Perceptions of Changes in Family Well-Being Following Participation in a Camp: Experiences of Families With a Child With ASD. *Ther. Recreation J.* **2017**, *51*, 143-163, doi:10.18666/Trj-2017-V51-I2-8359.

36. Rickard, H.C.; Dinoff, M. Behavior change in a therapeutic summer camp: A follow-up study. *The Journal of Genetic Psychology: Research and Theory on Human Development* **1967**, *110*, 181-183, doi:10.1080/00221325.1967.10533730.
37. Ward, T.; Goldingay, S.; Parson, J. Evaluating a supported nature play programme, parents' perspectives. *Early Child Dev Care* **2019**, *189*, 270-283, doi:10.1080/03004430.2017.1317764.
38. Zimmerman, H.T.; McClain, L.R. Exploring the outdoors together: Assessing family learning in environmental education. *Studies in Educational Evaluation* **2014**, *41*, 38-47.
39. Ashurst, K.L.; Smith, L.W.; Little, C.A.; Frey, L.M.; Werner-Wilson, T.A.; Stephenson, L.; Werner-Wilson, R.J. Perceived outcomes of military-extension adventure camps for military personnel and their teenage children. *American Journal of Family Therapy* **2014**, *42*, 175-189.
40. Ashurst, K.; Weisenhorn, D.; Atkinson, T. Extension military parent-teen camp experiences: Familyresilience building in action. *Journal of Extension* **2020**, *58*, 1-7.
41. Bettmann, J.E.; Russell, K.C.; Parry, K.J. How substance abuse recovery skills, readiness to change and symptom reduction impact change processes in wilderness therapy participants. *Journal of Child and Family Studies* **2013**, *22*, 1039-1050, doi:10.1007/s10826-012-9665-2.
42. Burdsal, C.; Buel, C.L. A short term community based early stage intervention program for behavior problem youth. *Journal of Clinical Psychology* **1980**, *36*, 226-241.
43. Harper, N.J.; Russell, K.C. Family involvement and outcome in adolescent wilderness treatment: A mixed-methods evaluation. 2008.
44. Huff, C.; Widmer, M.; McCoy, K.; Hill, B. The influence of challenging outdoor recreation on parent-adolescent communication. *Ther. Recreation J.* **2003**, *37*, 18-37.
45. Johnson, E.G.; Davis, E.B.; Johnson, J.; Pressley, J.D.; Sawyer, S.; Spinazzola, J. The effectiveness of trauma-informed wilderness therapy with adolescents: A pilot study. *Psychological trauma : theory, research, practice and policy* **2020**, *12*, 878-887.
46. Liermann, K.; Norton, C.L. Enhancing family communication: Examining the impact of a therapeutic wilderness program for struggling teens and parents. *Contemporary Family Therapy: An International Journal* **2016**, *38*, 14-22, doi:10.1007/s10591-015-9371-5.
47. McPeake, J.D.; Kennedy, B.; Grossman, J.; Beaulieu, L. Innovative adolescent chemical dependency treatment and its outcome: A model based on Outward Bound programming. *Journal of Adolescent Chemical Dependency* **1991**, *2*, 29-57.
48. Ramshini, M.; Hassanzadeh, S.; Afrooz, G.; Razini, H.H. The effect of family-centered nature therapy on interactions between parent and child with autism spectrum disorder. *Iranian Rehabilitation Journal* **2018**, *16*, 379-386, doi:10.32598/irj.16.4.379.
49. Russell, K.C. Adolescent substance-use treatment: Service delivery, research on effectiveness, and emerging treatment alternatives. *Journal of Groups in Addiction & Recovery* **2008**, *2*, 68-96, doi:10.1080/15560350802081264.
50. Scholl, K.G.; McAvoy, L.H.; Rynders, J.E.; Smith, J.G. The Influence of an Inclusive Outdoor Recreation Experience on Families that Have a Child With a Disability. *Ther. Recreation J.* **2003**, *37*, 38-57.
51. Tucker, A.R.; Msw, M.P.A.; Hobson, J.; Karoff, M.; Gass, M.A. Outdoor Behavioral Healthcare: Its Impact on Family Functioning. 2016.
52. Bradbury-Jones, C.; Aveyard, H.; Herber, O.R.; Isham, L.; Taylor, J.; O'Malley, L. Scoping reviews: the PAGER framework for improving the quality of reporting. *International Journal of Social Research Methodology* **2021**, 1-14, doi:10.1080/13645579.2021.1899596.
